# Supplementary material for: Music in noise recognition: An EEG study of listening effort in cochlear implant users and normal hearing controls
Source: PLoS One. 2023 Aug 10;18(8):e0288461. doi: 10.1371/journal.pone.0288461 (PMC10414671; doi:10.1371/journal.pone.0288461)
Supplement: S1 Annex — (DOCX) [file pone.0288461.s001.docx]

Annex: Musical Excerpts employed in the study

| Selected Stimuli | Emotion |
| --- | --- |
| Beethoven_Piano Concert 4(III mvt) a | Happy |
| Beethoven_Piano Concert 4(III mvt)b | Happy |
| Beethoven_Symphony 3 mov 3 | Happy |
| Beethoven_Synphony 6(III mov) | Happy |
| Haendel_Utrecht's Te Deum | Happy |
| Mozart_Piano Concert n 27 (III mov) | Happy |
| Ravel_Tombeau de Couperin (Rigaudon) | Happy |
| Verdi_La Traviata (Brindisi) | Happy |
| Albinoni_Adagio | Sad |
| Brahms_Piano Concerto 4 | Sad |
| Chopin_Nocturne Op 27 n1 | Sad |
| Grieg_Peer Gynt Suite 2 | Sad |
| Mozart_Piano Concert n 23-(II mov) | Sad |
| Ravel_Concert Sol (II mov) | Sad |
| Rodrigo_Concerto de Aranjuez | Sad |
| Schubert_String Quartet n 14 (II mov) | Sad |
