# Supplementary figures and images for: Music in noise recognition: An EEG study of listening effort in cochlear implant users and normal hearing controls
Source: PLoS One. 2023 Aug 10;18(8):e0288461. doi: 10.1371/journal.pone.0288461 (PMC10414671; doi:10.1371/journal.pone.0288461)

Annex: Clinical characteristics of CI users


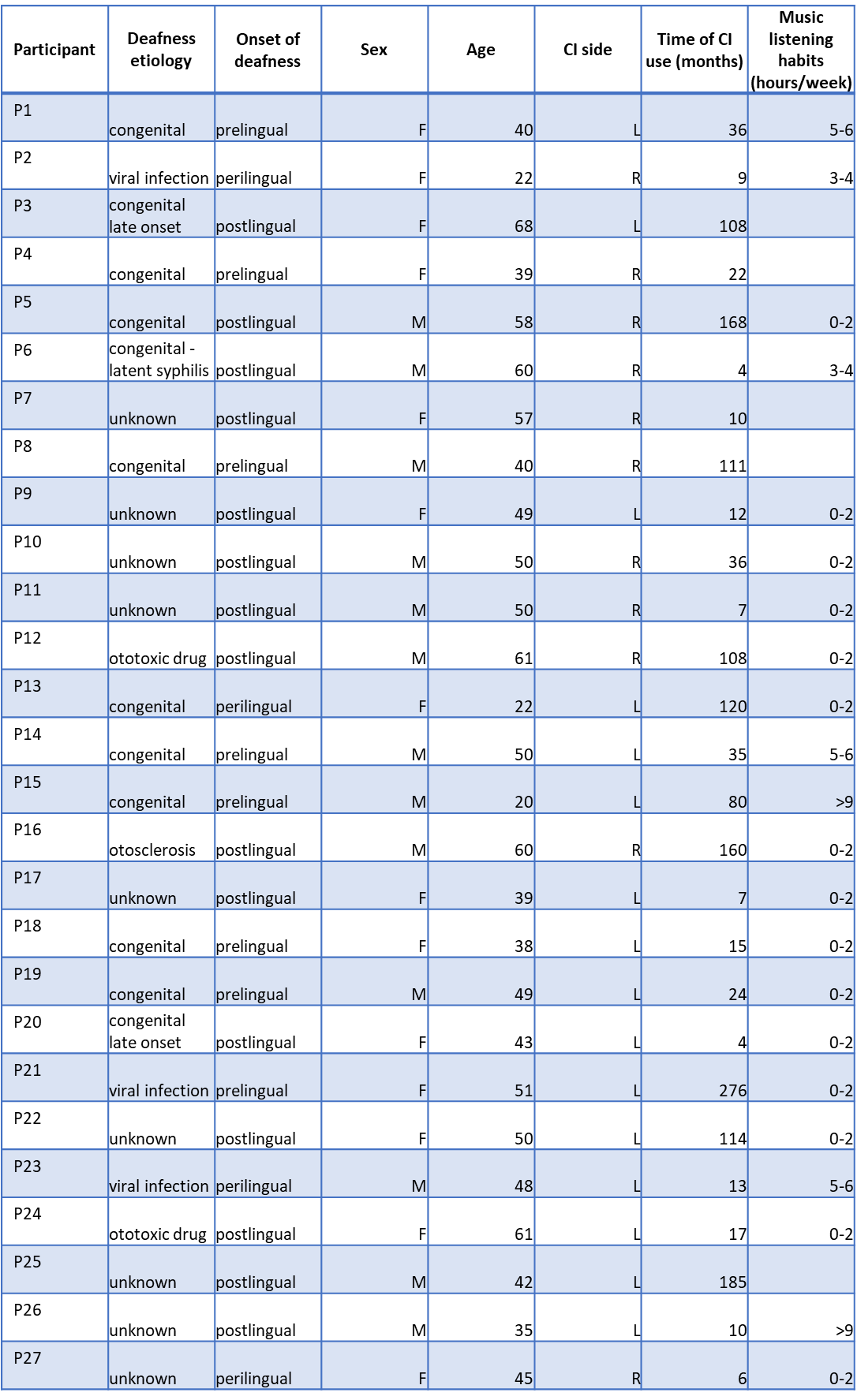

Supplement: S2 Annex — (DOCX) [file pone.0288461.s002.docx]
