# Supplementary material for: Music in noise recognition: An EEG study of listening effort in cochlear implant users and normal hearing controls
Source: PLoS One. 2023 Aug 10;18(8):e0288461. doi: 10.1371/journal.pone.0288461 (PMC10414671; doi:10.1371/journal.pone.0288461)
Supplement: S1 File — (PDF) [file pone.0288461.s003.pdf]

Correct Responses	
UCI	NH
83,33333	62,5
91,66667	87,5
70,83333	100
58,33333	91,66667
95,83333	91,66667
79,16667	100
70,83333	100
45,83333	100
70,83333	100
50	91,66667
20,83333	
37,5	
62,5	
54,16667	
20,83333	
79,16667	
75	
87,5	
45,83333	
100	
29,16667	
91,66667	
95,83333	
79,16667	
45,83333	
58,33333	
100	

	Correct Responses	
Group	Happy	Sad
UCI	91,66667	75
UCI	100	83,33333
UCI	66,66667	75
UCI	50	66,66667
UCI	100	91,66667
UCI	83,33333	75
UCI	41,66667	100
UCI	58,33333	33,33333
UCI	100	41,66667
UCI	100	0
UCI	33,33333	8,333333
UCI	41,66667	33,33333
UCI	75	50
UCI	91,66667	16,66667
UCI	25	16,66667
UCI	100	58,33333
UCI	91,66667	58,33333
UCI	91,66667	83,33333
UCI	75	16,66667
UCI	100	100
UCI	50	8,333333
UCI	91,66667	91,66667
UCI	91,66667	100
UCI	83,33333	75
UCI	50	41,66667
UCI	50	66,66667
UCI	100	100
NH	100	25
NH	91,66667	83,33333
NH	100	100
NH	100	83,33333
NH	100	83,33333
NH	100	100
NH	100	100
NH	100	100
NH	100	100
NH	100	83,33333

	Reactio
Group	Quiet
UCI	1714,875
UCI	1196,5
UCI	759,125
UCI	1510,25
UCI	371,875
UCI	1323,5
UCI	1737,75
UCI	2005,75
UCI	1559,875
UCI	1001,5
UCI	1752,5
UCI	4230
UCI	1193
UCI	1060
UCI	1399,625
UCI	1197,375
UCI	1187,875
UCI	1672,375
UCI	1332,25
UCI	1276,875
UCI	1450
UCI	1706,5
UCI	3285,375
UCI	1316,75
UCI	2817,75
UCI	3084,375
UCI	1419,375
NH	2321,875
NH	1368,375
NH	1709,5
NH	1305
NH	1369,375
NH	1062,625
NH	1639,625
NH	2175,625
NH	1336,875
NH	1547,375

n Times			Difficulty			
SNR10	SNR5	Group	% Easy	% Medium	%_Difficult	Group
1556,125	2532,5	UCI	37,5	29,16667	33,33333	UCI
1382,25	1333,75	UCI	100	0	0	UCI
883,75	754,25	UCI	66,66667	33,33333	0	UCI
2516,625	3070,25	UCI	54,16667	37,5	8,333333	UCI
406,625	676,5	UCI				UCI
1420,75	1466,75	UCI				UCI
3590,125	2696	UCI	100	0	0	UCI
2064,375	1943,625	UCI	0	100	0	UCI
2088,25	1765	UCI	95,83333	4,166667	0	UCI
1435,5	1861,375	UCI				UCI
2204,25	3138,625	UCI				UCI
3864,875	3789,25	UCI	29,16667	54,16667	16,66667	UCI
1752,625	1415,25	UCI	0	100	0	UCI
1392,75	2603	UCI				UCI
3179,875	2982,5	UCI				UCI
1644,875	1594	UCI	87,5	12,5	0	UCI
1628,875	1919,5	UCI	29,16667	29,16667	41,66667	UCI
1682,75	2549	UCI				UCI
1209	1576,375	UCI				UCI
1378,625	1419	UCI	83,33333	12,5	4,166667	UCI
1746,25	1759,25	UCI	0	66,66667	33,33333	UCI
1982,625	1845,5	UCI				UCI
3851,375	1871,375	UCI	83,33333	12,5	4,166667	UCI
955,625	1726,625	UCI	50	50	0	UCI
2096	2882	UCI	83,33333	12,5	4,166667	UCI
1625,75	2150,5	UCI	66,66667	20,83333	12,5	UCI
1390,25	1560,875	UCI	100	0	0	UCI
1710,125	2375,375	NH	100	0	0	NH
1214,625	1219	NH	25	54,16667	20,83333	NH
1527,125	1746,5	NH	87,5	12,5	0	NH
1402,5	1362,375	NH	91,66667	8,333333	0	NH
1689,625	1451,75	NH	41,66667	41,66667	16,66667	NH
1189,875	1286,375	NH	41,66667	37,5	20,83333	NH
1675,625	1969,5	NH	95,83333	4,166667	0	NH
2138,25	2214,25	NH	100	0	0	NH
1430,625	1510,625	NH	87,5	12,5	0	NH
2004,5	1711,875	NH	87,5	12,5	0	NH

%_Quiet_E	%_SNR10_	%_SNR5_E	%_Quiet_M	%_SNR10_	%_SNR5_M	%_Quiet_D	%_SNR10_	%_SNR5_Difficult
75	37,5	0	25	25	37,5	0	25	75
100	100	100	0	0	0	0	0	0
87,5	62,5	50	12,5	37,5	50	0	0	0
50	50	62,5	37,5	50	25	12,5	0	12,5
100	100	100	0	0	0	0	0	0
0	0	0	100	100	100	0	0	0
100	100	87,5	0	0	12,5	0	0	0
37,5	37,5	12,5	37,5	50	75	25	12,5	12,5
0	0	0	100	100	100	0	0	0
100	87,5	75	0	12,5	25	0	0	0
50	12,5	25	12,5	50	25	37,5	37,5	50
100	87,5	62,5	0	12,5	25	0	0	12,5
0	0	0	62,5	87,5	50	37,5	12,5	50
75	87,5	87,5	12,5	12,5	12,5	12,5	0	0
100	25	25	0	75	75	0	0	0
100	87,5	62,5	0	12,5	25	0	0	12,5
62,5	75	62,5	37,5	0	25	0	25	12,5
100	100	100	0	0	0	0	0	0
100	100	100	0	0	0	0	0	0
25	37,5	12,5	75	50	37,5	0	12,5	50
100	87,5	75	0	12,5	25	0	0	0
87,5	87,5	100	12,5	12,5	0	0	0	0
37,5	50	37,5	37,5	37,5	50	25	12,5	12,5
100	12,5	12,5	0	75	37,5	0	12,5	50
87,5	100	100	12,5	0	0	0	0	0
100	100	100	0	0	0	0	0	0
100	100	62,5	0	0	37,5	0	0	0
100	100	62,5	0	0	37,5	0	0	0

Alpha F8 SNR sub Quiet	
UCI	NH
0,0272895	-0,045138
-0,036302	-0,044896
0,0727422	-0,013157
0,0349555	-0,009398
0,0436842	-0,07076
0,0243282	-0,069094
-0,121842	-0,057472
0,1066562	-0,136091
-0,06106	-0,006975
0,0024742	-0,01228
-0,034431	
-0,052553	
0,013323	
-0,02554	
0,0011358	
0,0025829	
0,0670804	
0,0027084	
-0,133549	
0,0132962	
0,0501868	
0,0145914	
-0,011456	
-0,119543	
0,0037156	
0,0230051	
0,0002223	

Parietal Alpha SNR sub Quiet	
UCI	NH
0,023302231	-0,02419334
0,002094288	-3,8814E-05
0,037482395	-0,02120406
0,110776436	-0,02633842
-0,08096023	-0,02424793
0,001188358	-0,06136151
0,043680382	-0,04721521
0,016840795	-0,13823522
-0,06685486	0,00254774
-0,03906034	0,015892247
-0,08637316	
-0,02526865	
0,014720573	
0,067039197	
-0,02872539	
0,030928503	
-0,04913223	
-0,02419586	
-0,00341204	
-0,00145074	
-0,00179827	
0,039024455	
-0,03325958	
0,090452511	
0,030460513	
0,034570873	
-0,0169009	

Group	%_Liked
UCI	75
UCI	62,5
UCI	33,33333
UCI	37,5
UCI	
UCI	
UCI	8,333333
UCI	54,16667
UCI	62,5
UCI	
UCI	
UCI	37,5
UCI	29,16667
UCI	
UCI	
UCI	20,83333
UCI	50
UCI	
UCI	
UCI	41,66667
UCI	79,16667
UCI	
UCI	83,33333
UCI	37,5
UCI	20,83333
UCI	8,333333
UCI	50
NH	66,66667
NH	75
NH	66,66667
NH	62,5
NH	62,5
NH	75
NH	54,16667
NH	100
NH	33,33333
NH	58,33333

Pleasantness							
%_Indiffer	%_Disliked	UCI_Liked	NH_Liked	UCI_Indiff	NH_Indiffe	UCI_Dislik	NH_Dislike
4,166667	20,83333	75	66,66667	4,166667	33,33333	20,83333	0
29,16667	8,333333	62,5	75	29,16667	25	8,333333	0
29,16667	37,5	33,33333	66,66667	29,16667	33,33333	37,5	0
12,5	50	37,5	62,5	12,5	8,333333	50	29,16667
			62,5		20,83333		16,66667
			75		25		0
20,83333	70,83333	8,333333	54,16667	20,83333	8,333333	70,83333	37,5
45,83333	0	54,16667	100	45,83333	0	0	0
37,5	0	62,5	33,33333	37,5	29,16667	0	37,5
			58,33333		41,66667		0
25	37,5	37,5		25		37,5	
54,16667	16,66667	29,16667		54,16667		16,66667	
79,16667	0	20,83333		79,16667		0	
12,5	37,5	50		12,5		37,5	
29,16667	29,16667	41,66667		29,16667		29,16667	
0	20,83333	79,16667		0		20,83333	
16,66667	0	83,33333		16,66667		0	
41,66667	20,83333	37,5		41,66667		20,83333	
29,16667	50	20,83333		29,16667		50	
50	41,66667	8,333333		50		41,66667	
45,83333	4,166667	50		45,83333		4,166667	
33,33333	0						
25	0						
33,33333	0						
8,333333	29,16667						
20,83333	16,66667						
25	0						
8,333333	37,5						
0	0						
29,16667	37,5						
41,66667	0						

Period of CI use (months)
36
9
108
22
168
4
10
111
12
36
7
108
120
35
80
160
7
15
24
4
276
114
13
17
185
10
6
