# Supplementary material for: Music in noise recognition: An EEG study of listening effort in cochlear implant users and normal hearing controls
Source: PLoS One. 2023 Aug 10;18(8):e0288461. doi: 10.1371/journal.pone.0288461 (PMC10414671; doi:10.1371/journal.pone.0288461)
Supplement: Attachment — Submitted filename: Response to Reviewers_v2.pdf [file pone.0288461.s005.pdf]

REBUTTAL LETTER

Dear Editor, we thank for your final comments, please find the response to the following points raised:

Please remove all comentaries, ensure that all figures have their axis names and provide a clean version of the manuscript.

We apologise for the mistakes. We added axis title wherever it was not already specified and provided a clean version of the manuscript called “Manuscript_v2”

Clearly separate the text of figure legends from the text of the manuscript.

In the just mentioned revised manuscript we added a double space after captions.

Thanks again for your work and useful comments!
